# Percentage* of Adults Aged ≥65 Years Who Drank Four or More Alcoholic Drinks Per Week,^†^ by Sex and Age — National Health Interview Survey, United States, 2020^§^

**DOI:** 10.15585/mmwr.mm7133a5

**Published:** 2022-08-19

**Figure:**
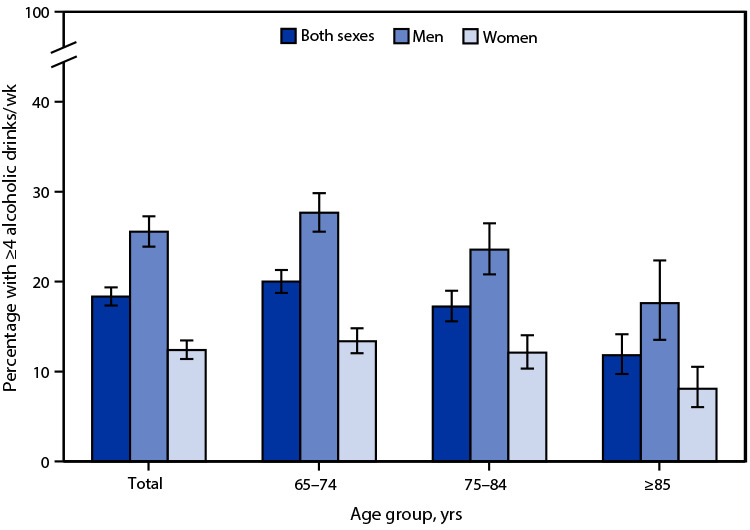
In 2020, 18.3% of adults aged ≥65 years reported drinking four or more alcoholic drinks per week. Among adults aged ≥65 years, men were more likely (25.6%) than women (12.4%) to have four or more drinks. Percentages of those having four or more drinks were higher among men than women for the following age groups: 65–74 years (27.7% versus 13.4%), 75–84 years (23.6% versus 12.1%) and ≥85 years (17.6% versus 8.1%). Among both men and women, the percentage of adults aged ≥65 years who drank four or more alcoholic drinks per week decreased as age increased, from 20.0% for those aged 65–74 years to 11.8% for those aged ≥85 years.

**Source:** National Health Interview Survey, 2020. https://www.cdc.gov/nchs/nhis.htm

For more information on this topic, CDC recommends the following link: https://www.cdc.gov/alcohol/index.htm

